# Supplementary material for: Vaccination has minimal impact on the intrahost diversity of H3N2 influenza viruses
Source: PLoS Pathog. 2017 Jan 31;13(1):e1006194. doi: 10.1371/journal.ppat.1006194 (PMC5302840; doi:10.1371/journal.ppat.1006194)

S1 Figure: Pre-season hemagglutination inhibition (HAI, A-C) and neuraminidase inhibition (NAI, D) titers against that season's vaccine strain for all individuals in this study. (A) HAI titers for individuals from the 2004-2005 season. (B) HAI titers for individuals from the 2005-2006 season. (C) HAI titers for individuals from the 2007-2008 season. (D) NAI titers for individuals from the 2007-2008 season. IIV, inactivated influenza vaccine; LAIV, live attenuated influenza vaccine. Dotted line, geometric mean.

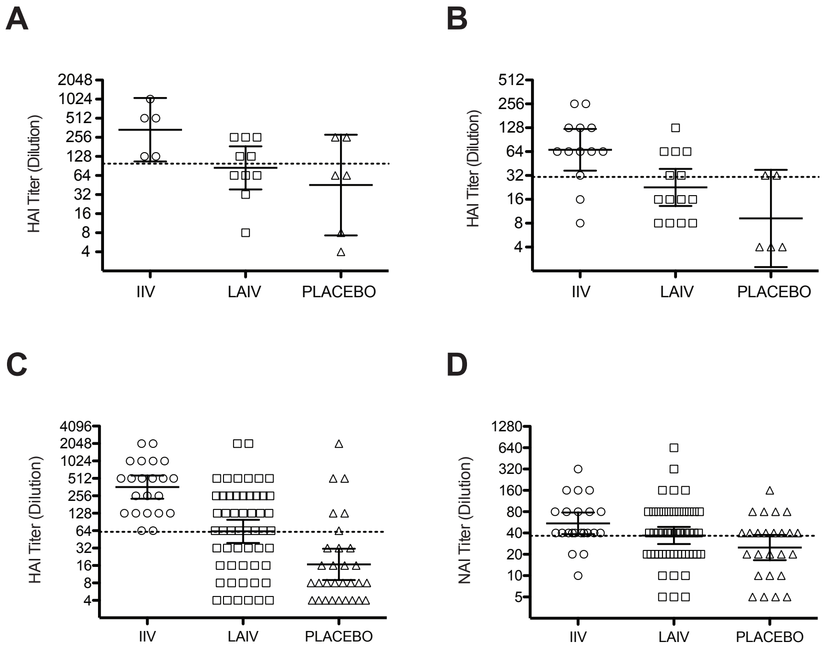

Supplement: S1 Fig — (PDF) [file ppat.1006194.s001.pdf]
