# Supplementary material for: Vaccination has minimal impact on the intrahost diversity of H3N2 influenza viruses
Source: PLoS Pathog. 2017 Jan 31;13(1):e1006194. doi: 10.1371/journal.ppat.1006194 (PMC5302840; doi:10.1371/journal.ppat.1006194)

S2 Figure: Genome copy number per µl viral transport media (y-axis) as determined by RT-qPCR for samples from 2004-2005 (left) and 2005-2006 (right) seasons by treatment group. IIV, inactivated influenza vaccine; LAIV, live attenuated influenza vaccine.

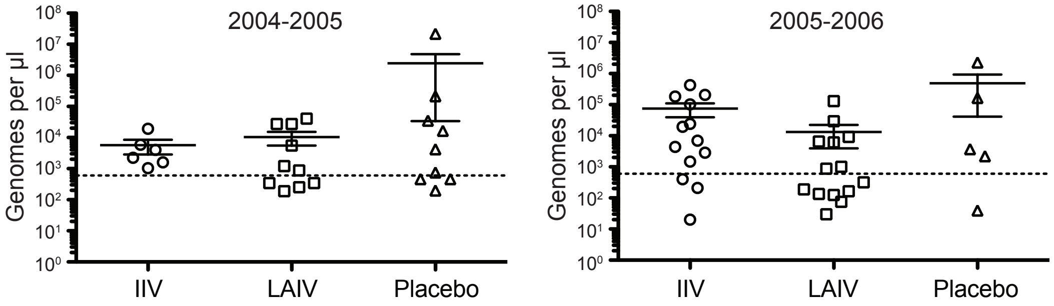

Supplement: S2 Fig — (PDF) [file ppat.1006194.s002.pdf]
