# Supplementary material for: Vaccination has minimal impact on the intrahost diversity of H3N2 influenza viruses
Source: PLoS Pathog. 2017 Jan 31;13(1):e1006194. doi: 10.1371/journal.ppat.1006194 (PMC5302840; doi:10.1371/journal.ppat.1006194)

S3 Figure: Sequence coverage for all samples in indicated seasons. The x-axis denotes each gene segment with bin width of 100 bases, while the y-axis shows average coverage per base within the bin. Boxplots are mean ± 1.5 interquartile range for samples within a season.

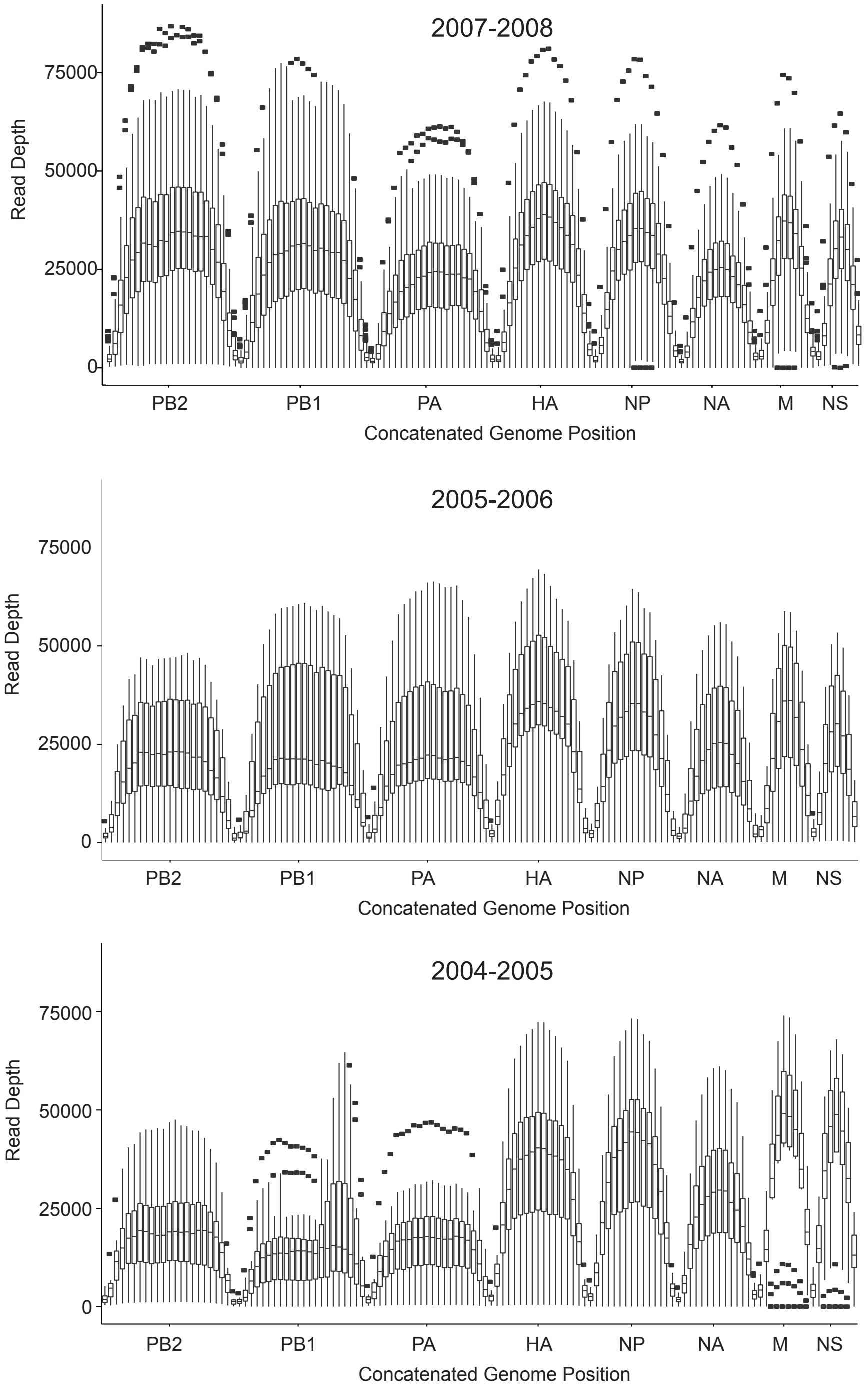

Supplement: S3 Fig — (PDF) [file ppat.1006194.s003.pdf]
