# Supplementary material for: Vaccination has minimal impact on the intrahost diversity of H3N2 influenza viruses
Source: PLoS Pathog. 2017 Jan 31;13(1):e1006194. doi: 10.1371/journal.ppat.1006194 (PMC5302840; doi:10.1371/journal.ppat.1006194)

S4 Figure: Maximum likelihood phylogenetic trees of consensus sequences for (A) HA, 2004-2005 (B) NA, 2004-2005 (C) HA 2005-2006 (D) NA 2005-2006 HA with tips coded by vaccine status and pre-season HAI (blue >40, magenta <40). Black, no data (note no NAI data). Outgroups are A/Wyoming/03/2003 (A,B) and A/California/7/2004 (C,D). Bootstrap values (n=1000 bootstraps) are shown and nodes with bootstrap values <50 are collapsed for easier visualization.

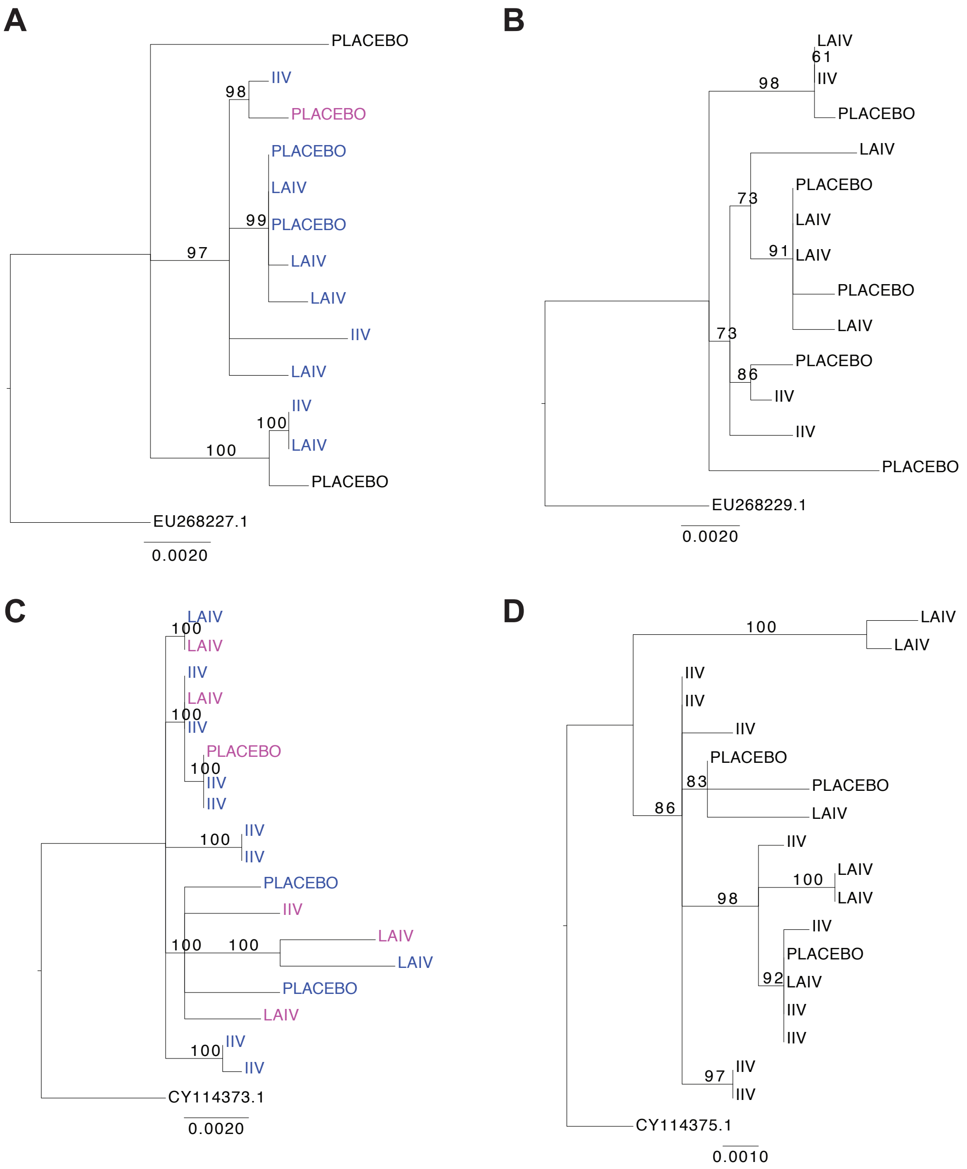

Supplement: S4 Fig — (PDF) [file ppat.1006194.s004.pdf]
