# Supplementary material for: Vaccination has minimal impact on the intrahost diversity of H3N2 influenza viruses
Source: PLoS Pathog. 2017 Jan 31;13(1):e1006194. doi: 10.1371/journal.ppat.1006194 (PMC5302840; doi:10.1371/journal.ppat.1006194)

S5 Figure: (A) Number of iSNV in HA per sample (y-axis) by HAI titer in that individual (x-axis). (B) Number of iSNV in NA per sample (y-axis) by NAI titer in that individual (x-axis).

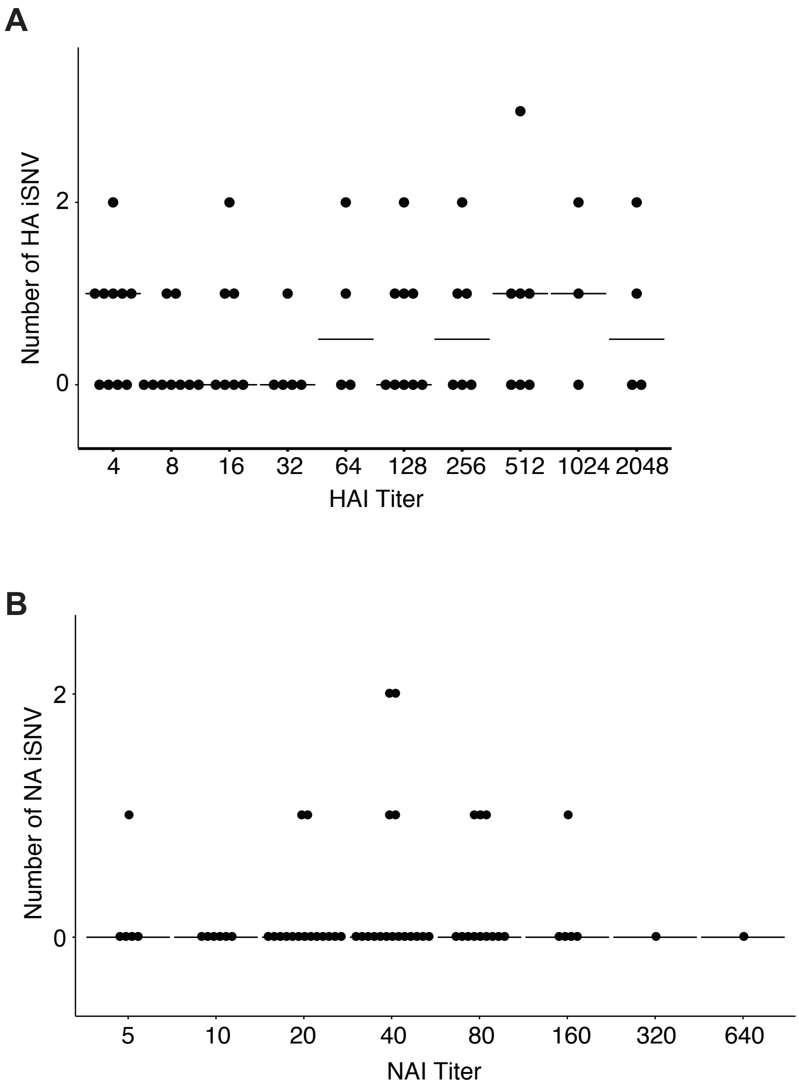

Supplement: S5 Fig — (PDF) [file ppat.1006194.s005.pdf]
