# Supplementary figures and images for: Vaccination has minimal impact on the intrahost diversity of H3N2 influenza viruses
Source: PLoS Pathog. 2017 Jan 31;13(1):e1006194. doi: 10.1371/journal.ppat.1006194 (PMC5302840; doi:10.1371/journal.ppat.1006194)

**A**

Number of iSNV

HAI Titer

> 40

< 40

**B**

Number of iSNV

NAI Titer

≥ 40

< 40

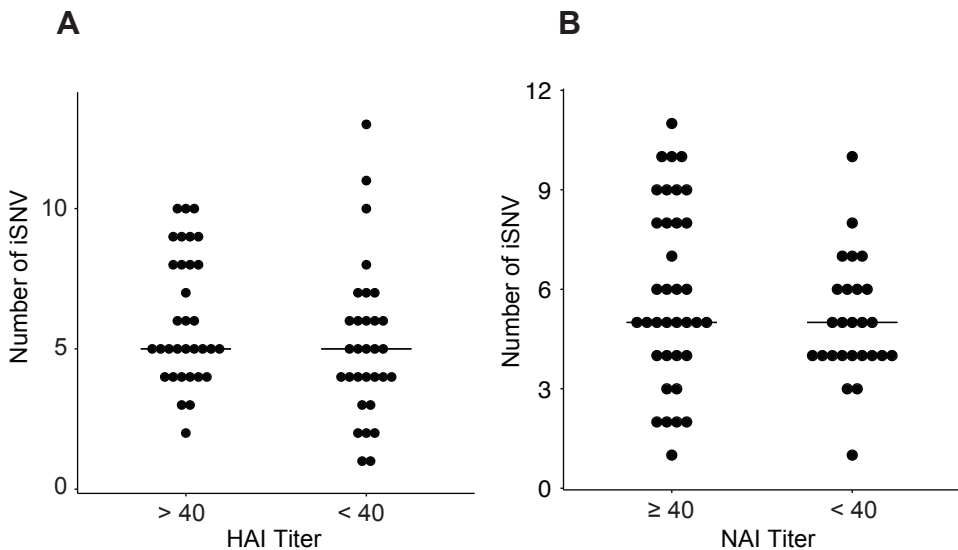

Supplement: S6 Fig — (PDF) [file ppat.1006194.s006.pdf]
