# Supplementary material for: Vaccination has minimal impact on the intrahost diversity of H3N2 influenza viruses
Source: PLoS Pathog. 2017 Jan 31;13(1):e1006194. doi: 10.1371/journal.ppat.1006194 (PMC5302840; doi:10.1371/journal.ppat.1006194)

S7 Figure: Histograms of iSNV frequency across entire cohort for the 2004-2005 (A) and 2005-2006 (C) seasons. Histograms of the number of specimens in which a given iSNV was found for the 2004-2005 (B) and 2005-2006 (D) seasons.

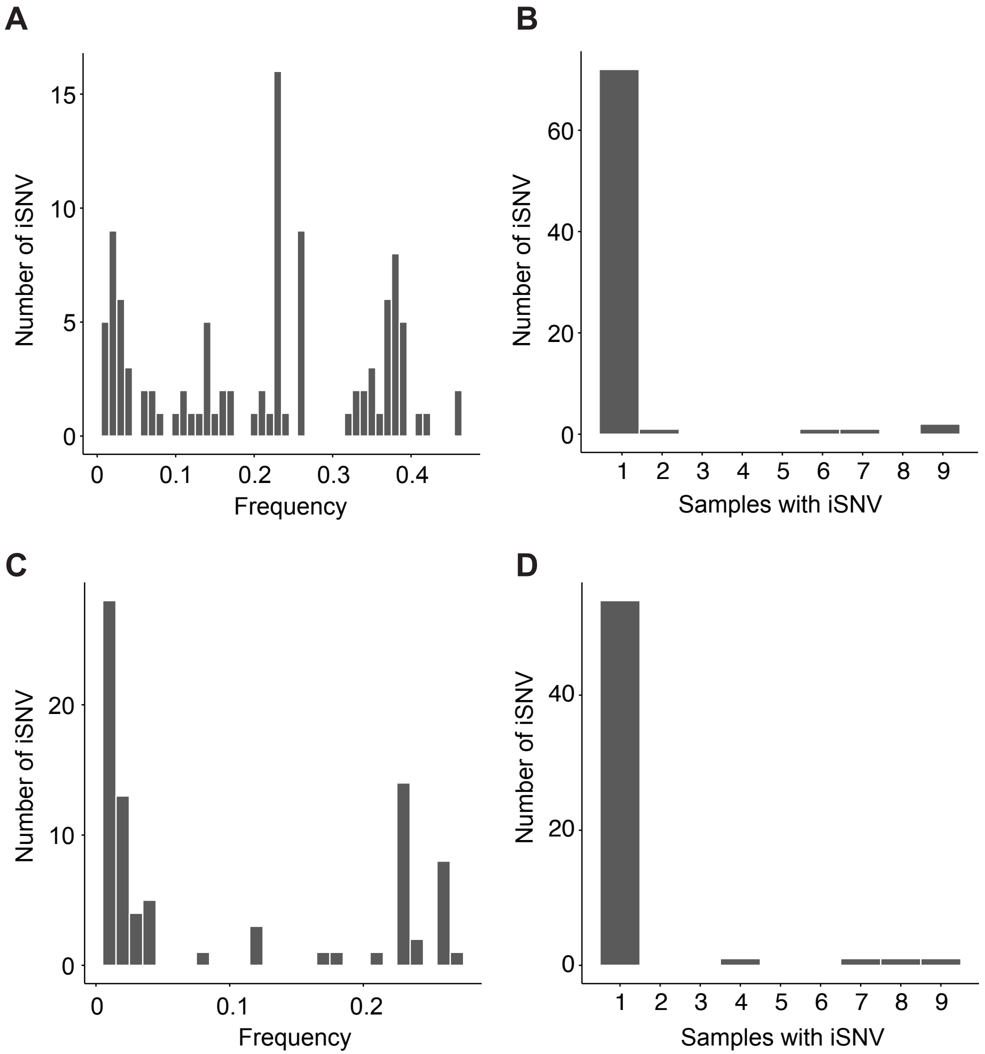

Supplement: S7 Fig — (PDF) [file ppat.1006194.s007.pdf]
