# Supplementary material for: Vaccination has minimal impact on the intrahost diversity of H3N2 influenza viruses
Source: PLoS Pathog. 2017 Jan 31;13(1):e1006194. doi: 10.1371/journal.ppat.1006194 (PMC5302840; doi:10.1371/journal.ppat.1006194)
Supplement: S1 Table — (DOCX) [file ppat.1006194.s008.docx]

**S1 Table: Variant Detection Specificity and Sensitivity by Copy Number.** Sensitivity and specificity for variant detection in experimental influenza populations with variants of known frequency and input titer. The benchmarking experiment and data are described in McCrone JT and Lauring AS, J. Virol. 90(15):6884, 2016.

| Copy Number^a^ | Variant Frequency | Sensitivity | Specificity |
| --- | --- | --- | --- |
|  |  |  |  |
| >10^5^ | 0.050 | 1.00 | >0.9999 |
|  | 0.020 | 0.85 | 0.9999 |
|  | 0.010 | 0.95 | 0.9995 |
|  | 0.005 | 0.35 | 0.9999 |
|  |  |  |  |
| 10^4^-10^5^ | 0.050 | 0.95 | 0.9999 |
|  | 0.020 | 0.90 | 0.9999 |
|  | 0.010 | 0.80 | 0.9998 |
|  | 0.005 | 0.40 | 0.9999 |
|  |  |  |  |
| 10^3^-10^4^ | 0.050 | 0.80 | >0.9999 |
|  | 0.020 | 0.45 | 0.9999 |
|  | 0.010 | 0.20 | 0.9997 |
|  | 0.005 | 0.10 | 0.9999 |
|  |  |  |  |
| ^a^ Per µl transport media | |  |  |
