# Supplementary material for: Vaccination has minimal impact on the intrahost diversity of H3N2 influenza viruses
Source: PLoS Pathog. 2017 Jan 31;13(1):e1006194. doi: 10.1371/journal.ppat.1006194 (PMC5302840; doi:10.1371/journal.ppat.1006194)
Supplement: S2 Table — (DOCX) [file ppat.1006194.s009.docx]

**S2 Table: Number of iSNV (mean ± interquartile range) by segment and treatment group and HAI titer**

| **2004-2005** | **Segment** | **IIV** | **LAIV** | **Placebo** | **HAI > 1:40** | **HAI < 1:40** |
| --- | --- | --- | --- | --- | --- | --- |
|  | 1 (PB2) | 1.67 ± (1.5) | 2 ± (0.5) | 3.33 ± (0.5) | 2 ± (1.25) | 4 ± (0) |
|  | 2 (PB1) | 6 ± (8.5) | 1 ± (0.5) | 0.67 ± (0.5) | 2.75 ± (1.25) | 1 ± (0) |
|  | 3 (PA) | 6.33 ± (9.5) | 0.5 ± (1) | 2 ± (1) | 2.75 ± (1) | 3 ± (0) |
|  | 4 (HA) | 0 ± (0) | 0 ± (0) | 0 ± (0) | 0 ± (0) | 0 ± (0) |
|  | 5 (NP) | 0.67 ± (0.5) | 0.75 ± (1.25) | 1 ± (1) | 0.88 ± (1.25) | 1 ± (0) |
|  | 6 (NA) | 1.33 ± (0.5) | 1 ± (0) | 1.67 ± (0.5) | 1.12 ± (0) | 2 ± (0) |
|  | 7 (M) | 2.67 ± (4) | 0 ± (0) | 0.33 ± (0.5) | 1 ± (0) | 1 ± (0) |
|  | 8 (NS) | 0.33 ± (0.5) | 0 ± (0) | 0 ± (0) | 0.12 ± (0) | 0 ± (0) |
|  |  |  |  |  |  |  |
| **2005-2006** | **Segment** | **IIV** | **LAIV** | **Placebo** | **HAI > 1:40** | **HAI < 1:40** |
|  | 1 (PB2) | 2.33 ± (0) | 4 ± (0) | 3 ± (0) | 2.33 ± (0) | 3.33 ± (0.5) |
|  | 2 (PB1) | 0.83 ± (1) | 1 ± (0) | 0.5 ± (0.5) | 0.83 ± (1) | 0.67 ± (0.5) |
|  | 3 (PA) | 0.33 ± (0.75) | 5 ± (0) | 0.5 ± (0.5) | 0.33 ± (0.75) | 2 ± (2.5) |
|  | 4 (HA) | 1.67 ± (0.75) | 5 ± (0) | 0 ± (0) | 1.67 ± (0.75) | 1.67 ± (2.5) |
|  | 5 (NP) | 0.5 ± (1) | 1 ± (0) | 2 ± (0) | 0.5 ± (1) | 1.67 ± (0.5) |
|  | 6 (NA) | 1.5 ± (0) | 1 ± (0) | 1.5 ± (0.5) | 1.5 ± (0) | 1.33 ± (0.5) |
|  | 7 (M) | 0.33 ± (0.75) | 0 ± (0) | 0.5 ± (0.5) | 0.33 ± (0.75) | 0.33 ± (0.5) |
|  | 8 (NS) | 0.5 ± (0) | 0 ± (0) | 0.5 ± (0.5) | 0.5 ± (0) | 0.33 ± (0.5) |
