# Supplementary material for: Vaccination has minimal impact on the intrahost diversity of H3N2 influenza viruses
Source: PLoS Pathog. 2017 Jan 31;13(1):e1006194. doi: 10.1371/journal.ppat.1006194 (PMC5302840; doi:10.1371/journal.ppat.1006194)
Supplement: S3 Table — (DOCX) [file ppat.1006194.s010.docx]

**S3 Table: Nonsynonymous variants in HA**

| HA Region | Amino Acid Substitution | Variant Frequency | Vaccination Status | Pre-season HAI Titer |
| --- | --- | --- | --- | --- |
|  |  |  |  |  |
| HA1 | G49S | 0.030 | Placebo | 16 |
| HA1 | D53N* | 0.012 | Placebo | 4 |
| HA1 | F79L | 0.040 | Placebo | 8 |
| HA1 | K208R* | 0.016 | Placebo | 8 |
| HA1 | K208R* | 0.015 | Placebo | 4 |
| HA1 | I214V* | 0.034 | Placebo | 512 |
| HA1 | K238N* | 0.029 | IIV | 1024 |
| HA1 | R269K | 0.079 | LAIV | 256 |
| HA1 | P273S* | 0.014 | LAIV | 2048 |
| HA1 | I274M | 0.010 | LAIV | 16 |
| HA1 | I278D* | 0.030 | LAIV | 128 |
| HA1 | V323I | 0.019 | Placebo | 512 |
|  |  |  |  |  |
| HA2 | A5S | 0.017 | LAIV | 64 |
| HA2 | Q27L | 0.021 | Placebo | 4 |
| HA2 | S29Y | 0.057 | IIV | 512 |
| HA2 | Q65L | 0.010 | IIV | 2048 |
| HA2 | A101T | 0.028 | LAIV | 16 |
| HA2 | N169H | 0.016 | IIV | 256 |

* Antigenic sites, as described in text and Figure 5
